# Preliminary Phytochemical Profile and Bioactivity of *Inga jinicuil* Schltdl & Cham. ex G. Don

**DOI:** 10.3390/plants11060794

**Published:** 2022-03-17

**Authors:** Ammy Joana Gallegos-García, Carlos Ernesto Lobato-García, Manasés González-Cortazar, Maribel Herrera-Ruiz, Alejandro Zamilpa, Patricia Álvarez-Fitz, Ma Dolores Pérez-García, Ricardo López-Rodríguez, Ever A. Ble-González, Eric Jaziel Medrano-Sánchez, Max R. Feldman, Alejandro Bugarin, Abraham Gómez-Rivera

**Affiliations:** 1 División Académica de Ciencias Básicas, Universidad Juárez Autónoma de Tabasco, Carretera Cunduacán-Jalpa Km. 0.5, Cunduacán 86690, Tabasco, Mexico; joana90102010@gmail.com (A.J.G.-G.); ricardo.lopezr@ujat.mx (R.L.-R.); ble_49@hotmail.com (E.A.B.-G.); ericsanz123@gmail.com (E.J.M.-S.); 2 Centro de Investigación Biomédica del Sur, Instituto Mexicano del Seguro Social, Argentina No. 1, Col. Centro, Xochitepec 62790, Morelos, Mexico; cibis_herj@yahoo.com.mx (M.H.-R.); azamilpa_2000@yahoo.com.mx (A.Z.); lola_as@yahoo.com.mx (M.D.P.-G.); 3 Laboratorio de Toxicología, Cátedra CONACyT-Universidad Autónoma de Guerrero, Av. Lázaro Cárdenas s/n. Col. La Haciendita, Chilpancingo 39070, Guerrero, Mexico; paty_fitz@hotmail.com; 4 Department of Chemistry and Physics, Florida Gulf Coast University, Fort Myers, FL 33965, USA; mrfeldman2018@eagle.fgcu.edu (M.R.F.); abugarin@fgcu.edu (A.B.)

**Keywords:** *Inga jinicuil*, phytochemical profile, HPLC-PDA, GC-MS, anti-inflammatory, antimicrobial

## Abstract

Several Mesoamerican cultures have used *Inga jinicuil* as traditional medicine for the treatment of gastrointestinal, inflammatory, and infectious issues. The aims of this contribution were to elucidate the phytochemical profile of the organic extracts from the bark and leaves of *I. jinicuil* and to assess the anti-inflammatory and antibacterial properties of these extracts. The preliminary chemical profile was determined by HPLC-PDA and GC-MS; the anti-inflammatory activity was evaluated with a mouse ear edema model, whereas the antibacterial activity was screened against several bacteria. The phytochemical profile of both organs (bark and leaves) of *I. jinicuil* led to the identification of 42 compounds, such as polyphenolic, flavonoids, triterpenes, prenol-type lipids, and aliphatic and non-aliphatic esters. This molecular diversity gave moderate anti-inflammatory activity (67.3 ± 2.0%, dichloromethane bark extract) and excellent antibacterial activity against *Pseudomona aeruginosa* and methicillin-resistant *Sthaphylococcus aureus* (MIC values of ˂3.12 and 50 µg/mL, respectively). These results contribute to the chemotaxonomic characterization and the rational use in traditional medicine of *Inga jinicuil* Schltdl & Cham. ex G. Don.

## 1. Introduction

Anti-inflammatory and antimicrobial properties have been attributed to a great diversity of plants used in traditional medicine, from which many commercial drugs have been developed [1]. These properties have been related to the presence of secondary metabolites such as tannins, terpenes, and flavonoids, among many others [2]. Currently, medicinal plants are a valuable alternative and, in agreement with the WHO strategies on complementary and traditional medicine, it is necessary to perform studies aimed at identifying their bioactive compounds and confirming their pharmacological activity in order to guarantee their safe, effective, and rational use [3].

Even though there are several alternatives for the treatment of inflammation, some anti-inflammatory drugs, such as aspirin, ketorolac, naproxen, or piroxicam, have adverse effects (e.g., the risk of developing intestinal bleeding) [2]; therefore, a constant search for new anti-inflammatory treatments is critical in order to achieve an increased pharmacological response with the lowest degree of unwanted side effects [4]. The rise of microbial strains resistant to current antibiotics similarly presses the medical field to find new, effective compounds. These issues have led to the search for alternatives derived from natural sources to help in either the prevention or treatment of infectious problems [5].

Related to the above statements, a promissory prospect is the tropical species *Inga jinicuil* Schltdl & Cham. ex G. Don, known in Mexico as “cuijinicuil”, “cuajicuil”, or “jinicuil”, and named in the Maya-Chontal language as “bujte”. This plant belongs to the *Leguminosae* family and is classified as a multi-purpose tree in Mesoamerican indigenous communities, where it is mainly used as a shade tree in agroecosystems for cocoa and coffee plantations [6,7]. It is also an ornamental tree present in many gardens, parks, and roads, and it is highly recommended for repopulating watersheds [6]. The cotton pulp that covers the seeds can be consumed fresh or used in jellies and drinks [8]. Some indigenous communities of the Maya-Chontal region in Mexico and in the Amazon boil the seeds in salt water and use it as an appetizer or complement in traditional stews [6,7]. The aerial parts are used for healing purposes for the treatment of parasitic and infectious problems [6,8]. A mixture of seeds and leaves is also used as both an antidiarrheal and antirheumatic remedy [6]; in rural communities of Veracruz and Tabasco, Mexico, this plant is also utilized for gastrointestinal diseases by taking an infusion made from the pod and bark [7,8].

There are few reports on the phytochemical and biological activity of the *Inga* genus. For instance, a recent report highlights the antibacterial activity of the organic extracts from the leaves of *I. semialata*, which had an inhibitory effect on the growth of *Staphylococcus aureus*, *Staphylococcus epidermidis*, *Micrococcus luteus*, and *Klebsiella pneumoniae* associated with recurrent infections; the analysis of the extracts revealed the presence of gallic acid, epicatechin, and rutin [9]. There is also a series of reports aimed at the phytochemical and pharmacological analysis of *I. edulis* and *I. laurina*, where antimicrobial and antioxidant activities have been associated mainly with the presence of phenolic compounds [10,11,12,13]. In the case of *I. edulis*, the dichloromethane extract from leaves exerts a moderate antibacterial activity (MIC 7.0 mg/mL) against two strains of *S. aureus*; whereas for *I. laurina*, its effect against some strains of aerobic and anaerobic micro-organisms has been reported. The chemical composition of the active extract was determined by GC-MS, finding terpenoids, fatty acids, and esters [10,11,12,13]. Despite the extensive use of *I. jinicuil* in traditional medicine in southeastern Mexico, only the antimicrobial activity of hexanic and chloroform extracts from the seeds has been reported. These extracts proved to be active against *S. aureus* 25,923 and *Listeria monocytogenes* 244, with an MIC of 100 µg/mL for each micro-organism [14]; however, to date, no studies have been found about the phytochemical composition of the bark and leaves of this plant, nor on the evaluation of its anti-inflammatory activity. Therefore, the objectives of this work were to analyze the phytochemical profile of organic extracts from the bark and leaves of *I. jinicuil* via chromatographic methods, to evaluate their anti-inflammatory activity in the phorbol ester (TPA)-induced mouse ear edema test, and to expose its antibacterial activity against strains of clinical importance.

## 2. Results and Discussion

The yield of the extracts from *Inga jinicuil* are shown in Table 1. Three types of extracts (in order of increasing polarity) were acquired from bark (Hexane **Ij-BH**, Dichloromethane **Ij-BD**, & Hydroalcoholic **Ij-BHac**) and three more from leaves (Hexane **Ij-LH**, Dichloromethane **Ij-LD**, & Hydroalcoholic **Ij-LHac**). It was found that, in general, the extracts from leaves provided higher yields when compared to bark extracts, with the hydroalcoholic extract from leaves being the one with the highest percentage.

### 2.1. HPLC and UV-Vis Spectra Analysis of Polar Extracts from Inga jinicuil

**Figure 1:**
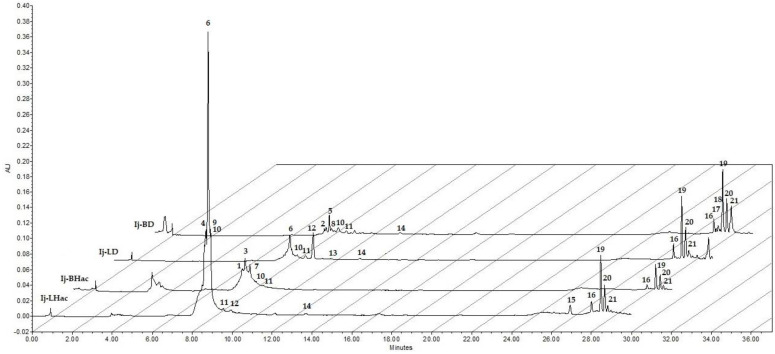
HPLC chromatograms of bark (**Ij-BD**, **Ij-BHac**) and leaf (**Ij-LD**, **Ij-LHac**) extracts. The peaks are numbered in ascending order according to their retention times (λ = 270 nm).

HPLC analysis helped to determine the presence of terpenic and flavonoid-type compounds in both the dichloromethane and hydroalcoholic extracts from *I. jinicuil*. The chromatograms of the four polar extracts (**Ij-LD**, **Ij-LHac**, **Ij-BD**, and **Ij-BHac**) are presented in Figure 1. A total of 21 peaks related to terpenic and flavonoid-type compounds were observed. Table 2 presents a summary of the following information: retention times, main absorption bands of the UV-Vis spectra, and the presence of each peak in the four extracts analyzed.

As seen in Figure 1, the peaks in the chromatograms can be differentiated into two main groups based on their retention time (*t*_R_): the first group consists of 14 peaks with *t*_R_ between 8 and 12 min, while the second group has 7 peaks with *t*_R_ from 26 to 29 min.

Based on the retention times and the absorption bands in the UV-Vis spectra (Figures S1–S4) of the peaks shown in Table 2, it was possible to perform a preliminary analysis of each of the metabolites present in the extracts by comparing those parameters with known standards and data from the literature. Accordingly, for Peak **1**, the observed absorption bands at 220.4, 261.6, and 294.7 nm were equal with those of the protocatechuic acid standard, which when analyzed in identical chromatographic conditions presented the same *t*_R_ (8.46 min). Since Peak **2** showed chromatographic behavior similar to **1** along with the analysis of the UV-Vis bands and the literature, it is inferred that it may be a derivative of protocatechuic acid [15,16].

Regarding Peaks **3** and **7**, when their *t*_R_ and UV-Vis absorption bands were compared with the gallic acid standard (*t*_R_ = 7.46 min, λ_max_ = 220.4, 272.2 nm), a good match was found in the UV-Vis spectrum; however, the differences in retention times suggested the presence of gallic acid derivatives [17,18].

The analysis of the UV-Vis bands for Peaks **4**, **6**, and **9** indicated that these compounds may be of the flavonoid type; this inference was strengthened when they were compared with an apigenin standard (*t*_R_ = 16.76 min, λ_max_ = 211, 267.5, 338.6 nm) and an identical match was found in their UV-Vis spectra. The differences in retention times led us to potentially consider these peaks as glycosylated analogs of this flavone [19,20].

For the case of Peak **5**, its UV-Vis pattern was comparable to those reported for lignane-type compounds. Similarly, Peak **8** presented characteristic bands associated with derivatives of coumaric acid [23]. On the other hand, for Peaks **12** and **18**, their UV-Vis spectra were characteristic of those reported for coumarin-type compounds [25,26,27].

Regarding the analysis of the UV-Vis spectra of Peaks **10**, **11**, **14**, and **16**, it was possible to associate them with previous reports for terpene derivatives [24]. Likewise, Peaks **13** and **17** were consistent with bibliographical data of epigallocatechin gallate derivatives [28], and Peak **15** may be associated with vanillic acid derivatives [29,30]. Finally, the absorption bands of Peaks **19**, **20**, and **21** were associated with salicylate derivatives [31].

Considering the above information, Peaks **10**, **11**, **16**, **19**, **20**, and **21**, attributed to terpenes and salicylates, were detected in the four extracts analyzed, whereas Peak **14**, which was also recognized as a terpene, was found in three extracts (absent in **Ij-BHac**). The two leaf extracts shared the presence of Peaks **6** and **12**, which were consistent with apigenin and coumarin derivatives, respectively. Despite this, as can be appreciated in Table 2, around 60% of the metabolites identified in the polar extracts of the bark and leaves of *Inga jinicuil* were only found in one extract.

It should be noted that, to date, no reports have been found on secondary metabolites present in bark or leaves from *I. jinicuil*, so this work represents a first approach for the phytochemical study of these organs of the plant. However, there are reports about the phytochemical composition for other species of the *Inga* genus, where the presence of a high content of polyphenols with an important antioxidant capacity has been demonstrated [31]. For *I. semialata* and *I. edulis*, the analysis of leaf extracts allowed the identification of compounds such as: epicatechin, apigenin C-di-hexoside, myricetin-*O*-hexose-deoxyhexose, myricetin-*O*-deoxyhexose, and vicenin-2 [9,32]. Likewise, other studies on leaf extracts from *I. edulis*, reported the presence of four triterpenes (lupeol, α-amirin, olean-18-ene acid, and frideline), three flavonoids, eight phenolic acids, an anthocyanin derived from delphinidin-3-glycoside, and a mixture of five acylated anthocyanins. It is important to highlight the fact that gallic acid, methyl gallate, protocatechuic acid, and quercetin were also identified [33]. For *I. laurina*, there is a presence of flavonoids 3-O-(2″-O-galloyl)-α-rhamnopyranoside and myricetin-3-rhamnoside in leaf extracts [19], as well as gallic acid, myricetin derivatives, quercetin glycoside, and glycoside myricetin-3-O-rhamnosid from ethanolic extracts of leaves from this plant [20].

In view of the above-mentioned studies, our preliminary assessment of the phytochemical profile of *Inga jinicuil* allowed the identification of a chemotaxonomic resemblance with other species of the same genus, since a shared presence of phenolic and terpenic compounds, such as gallates, protocatechuic acid, and its derivatives, as well as flavonoids such as apigenin, can be recognized. It should be emphasized that in published work, the phytochemical research reports on *I semialata*, *I. eludis*, and *I. laurina* refer mainly to polar leaf extracts, whereas the phytochemical analysis of bark has been oriented to non-polar extracts (as discussed below). Therefore, this report also contributes to the identification of secondary metabolites in polar extracts from this organ for a species of the *Inga* genus.

### 2.2. Chemical Profile of Hexane Extracts from Bark and Leaves of I. jinicuil by GC-MS

**Figure 2:**
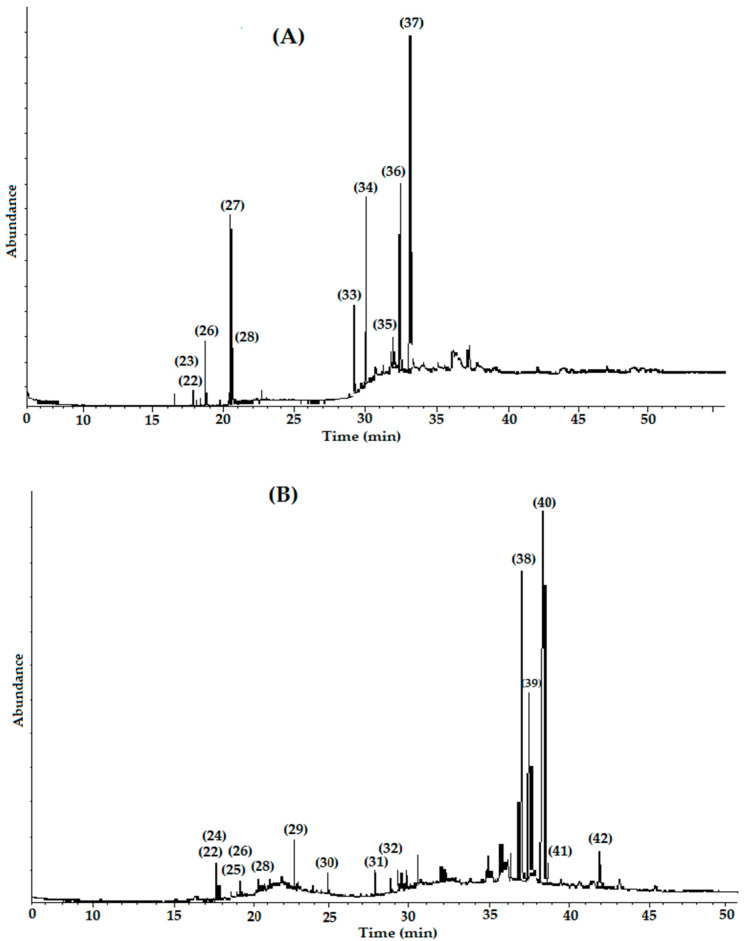
GC chromatograms of the hexanic extracts: (**A**) **Ij-BH** and (**B**) **Ij-LH**. The peaks are numbered in ascending order according to their retention times.

The analysis of the GC-MS chromatograms of **Ij-BH** and **Ij-LH** [Figure 2A,B] allowed the identification of 21 compounds, where 7 of them were only found in the bark extracts, 11 compounds only appeared in the analysis of the leaf extracts, and 3 werecommon to the extracts of both organs. Table 3 presents a list of the compounds detected arranged according to their elution order. The most abundant compounds detected for **Ij-BH** were prenol, α-tocopherol (relative abundance: 40.49%), and triterpene 24-methylenecycloartan-3-one (38.61%); these compounds represented approximately 80% of the content of this extract. For the **Ij-LH** extract, the triterpenes included lup-20 (29)-en-3-one (26.74%) and lupeol (16.44%), as well as the aliphatic compound hentriacontane (16.66%), all of which constituted nearly 60% of its metabolic content. The compounds identified in both extracts were hexadecanoic acid methyl ester, hexadecanoic acid ethyl ester, and octadecanoic acid methyl ester. It is worth mentioning that these three compounds were found in greater abundance in **Ij-BH**.

It is worth noting that this is the first report of a CG-MS analysis of hexanic extracts from *Inga jinicuil*. However, similar studies have been documented for other species of the *Inga* genus; such is the case for *I. edulis*, where triterpene compounds including lupeol and stigmasterol, as well as aliphatic compounds, have been identified from extracts of the bark and leaves [33,34]. Likewise, extracts from the bark and leaves of *I. laurina* have shown the presence of terpenes such as phytol, the aliphatic nonacosane, and esterified aliphatic acids [35], whereas in a hexanic fraction obtained from the leaves of *I. semialata*, the main compounds isolated were triterpenes, such as lupeol, α-amyrin, oleanolic acid, and friedelin [30]. In this report, the presence of esterified aliphatic acids was identified and, as in other species of the *Inga* genus, the presence of lupeol has been established. However, the following compounds: hentriacontane, α-tocopherol, lup-20 (29)-en-3-one and 24-methylenecycloartan-3-one, are reported for the first time for this genus.

### 2.3. Anti-Inflammatory Activity of Organic Extracts from Inga jinicuil

**Figure 3:**
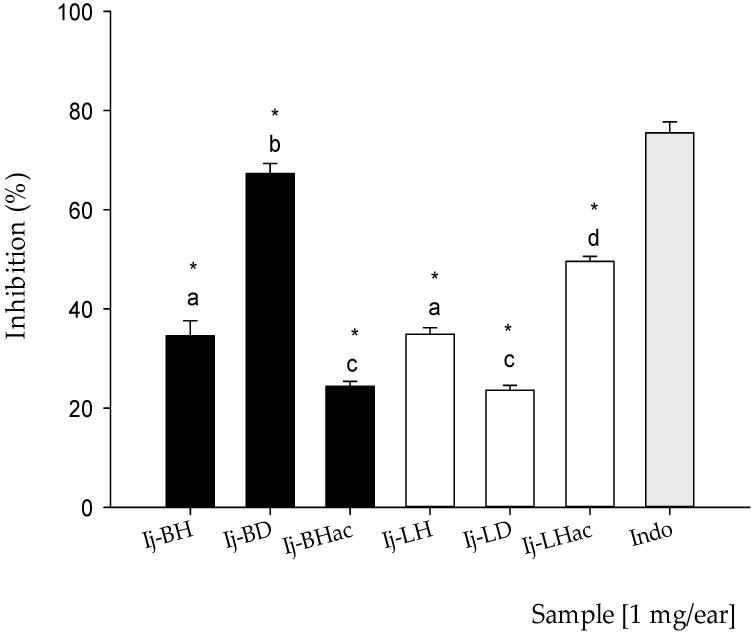
Percentage inhibition of inflammation (%) of **Ij-BH**, **Ij-BD**, **Ij-BHac**, **Ij-LH**, **Ij-LD**, and **Ij-LHac** extracts from *Inga jinicuil* and Indo (Indomethacin) in edema induced by TPA in mouse ear at 1.0 mg/ear. Values are presented as means ± standard error of the means (SEM). *n* = 5. ANOVA, with post-test Dunnet with * *p* ≤ 0.05 in comparison with Indo and Tukey test, where different letters indicate significant differences among them.

The results corresponding to the anti-inflammatory study of the organic extracts are presented in Figure 3. At the same dose of 1.0 mg/ear, all of the extracts showed anti-inflammatory activity. For the bark extracts, the percentages of inhibition were: **Ij-BH** 34.6 ± 3.0%, **Ij-BD** 67.3 ± 2.0%, and **Ij-BHac** 24.4 ±1.0%, and for leaf extracts, the corresponding percentages were: **Ij-LH** 34.9 ± 1.3%, **Ij-LD** 23.0 ± 1.0%, and **Ij-LHac** 49.6 ± 1.0%. For indomethacin (**Indo**), which was employed as the reference drug, the inhibition percentage was 75.5 ± 2.2%. As can be seen, the two extracts with the greatest anti-inflammatory activity were **Ij-BD** followed by **Ij-LHac**, and the statistical comparison between the anti-inflammatory activities of the extracts and the reference drug revealed significant differences (*p* < 0.05). No extract reached an effect equal to or greater than that of **Indo** (Indomethacin). However, the comparison using the Tukey test of the effect of the extracts and the reference drug showed that there were no significant differences (*p* < 0.05) between some of the extracts, such as **Ij-BH** compared to **Ij-LH** and **Ij-BHac** compared to **Ij-LD**.

Even when species such as *I. laurina*, *I. edulis*, *I. marginata*, and *I. jinicuil* are employed to treat stomach and inflammatory disorders in traditional medicine, few studies have been conducted to confirm their attributed pharmacological properties. However, recent reports have shown the presence of flavonoids and other phenolic compounds in several of these species that may be associated with pharmacological effects [36]. The present study represents a preliminary approach in the assessment of the anti-inflammatory activity of *I. jinicuil*, with the bark extracts exerting a more consistent effect and **Ij-BD** showing the highest activity. It is noteworthy to mention that the chemical profile of this extract showed the presence of salicylates, terpenoids, and derivatives of epigallocatechin gallate, as well as derivatives of protocatechuic and coumaric acids, which may be associated with its biological effect [24,28,31]. In the case of the extracts from leaves, **Ij-LHac** showed the best inhibitory effect, and the analysis of its metabolic content revealed the presence of polyphenolic compounds, terpenoids, coumarins, vanillic acid derivatives, and flavonoid-type compounds such as apigenin derivatives, all of which have reported anti-inflammatory effects [19,34,37]. Furthermore, previous reports regarding several of the metabolites present in both extracts have postulated an anti-inflammatory activity that proceeds via the inhibition of cyclooxygenase enzymes (COX) [38,39,40], which happens to be the known mechanism of the reference drug (**Indo**) [40]. Finally, it is important to mention that the two extracts with the highest activity have the presence of terpenes and salicylates in common; these compounds are recognized for their analgesic and anti-inflammatory effects [24,31].

### 2.4. Antibacterial Activity of Inga jinicuil Organic Extracts

The antibacterial activity of bark and leaf *I. jinicuil* extracts were evaluated on clinically important micro-organisms. As showed in Table 4, the three extracts of bark (**Ij-BH**, **Ij-BD**, and **Ij-BHac**) exhibited excellent activity against *Pseudomona aeruginosa* (**Pa**) and methicillin-resistant *Staphylococcus aureus* (**Sa1**), with MIC values of ˂3.12 and 50 µg/mL, respectively. Only **Ij-BH** showed activity against *Staphylococcus epidermidis* (**Se1**). Similarly, **Ij-LD** and **Ij-LHac** had good activity against *Pseudomona aeruginosa* (**Pa;** MIC ˂ 3.12 µg/mL), methicillin-resistant *Staphylococcus aureus* (**Sa1**; MIC = 50 µg/mL) and *Staphylococcus epidermidis* (**Se1**; MIC = 200 µg/mL).

The results obtained are interesting considering that in 2017 the WHO published a list of “priority pathogens” resistant to antibiotics, which include *Pseudomonas aeruginosa* (resistant to carbapenems) and *Staphylococcus aureus* (resistant to methicillin), emphasizing the urgent need for the search for new agents against these micro-organisms [5].

The antibacterial activity of bark and leaf extracts against *Pseudomona aeruginosa* (**Pa**) and methicillin-resistant *Staphylococcus aureus* (**Sa1**) can be attributed to the presence of several secondary metabolites: hentriacontane and α-tocopherol in **Ij-BH,** and polyphenols, flavonoids, and terpenoids in both **Ij-BD** and **Ij-BHac** [41]. Special attention may be paid to the presence of gallate and coumarin derivatives, since their antibacterial mechanism has been described at the cell membrane level by repressing the transport system of proteins and inhibiting the biofilm formation in clinical strains of **Sa1** [17,42,43]. Furthermore, coumarin derivatives are considered as potential antibacterial agents that act as inhibitors to several binding proteins of **Sa1** and potential competitive inhibitors of the DNA-gyrase [44,45]. It is worth noting that the chemical moiety responsible for the antibacterial activity of coumarins is the basic structure of benzopyrone, which resembles the structure of benzopyridone present in antibacterial drugs derived from quinolone [44,45]. Therefore, the wide range of chemical structures found in these extracts may represent a potential source of molecular templates for new antibacterial drugs.

## 3. Materials and Methods

### 3.1. Plant Material and Extraction of Inga jinicuil

Aerial parts of *Inga jinicuil* Schltdl & Cham. ex G. Don were collected in July 2019, in Libertad, Cunduacán, Tabasco, Mexico (10 m.a.s.l., latitude 18°10′53.06 N, longitude 93°22′28.13 W). A specimen was deposited at the Herbarium of the Academic Division of Biological Sciences of the Universidad Juárez Autónoma de Tabasco for its taxonomic identification (voucher number: 36576).

Plant material was dried at room temperature in the dark for 72 h, with drying and spraying in Pulvex MP300 milled (4–6 mm). The extracts were obtained by maceration with *n*-hexane, dichloromethane, and a 60:40 ethanol:water mixture 1:4; the maceration procedure was performed three times for each solvent in order to ensure an exhaustive extraction. These extracts were filtered, concentrated in a rotary evaporator (Heidolph G3, Schwabach, Germany), and then lyophilized (Heto Drywinner DW3) to give the bark (*n*-Hexane **Ij-BH**, Dichloromethane **Ij-BD**, Hydroalcoholic **Ij-BHac**) and leaf extracts (*n*-Hexane **Ij-LH**, Dichloromethane **Ij-LD**, Hydroalcoholic **Ij-LHac**).

### 3.2. HPLC Analysis

Chromatographic analysis was carried out in a Waters 2695 separation module system with a Waters 2695 photodiode matrix detector and Empower Pro software (Waters Corporation, Milford, MA, USA). Chemical separation was performed using a Supelcosil LC-F column (4.6 mm × 250 mm i.d., particle size 5 μm) (Sigma-Aldrich, Bellefonte, PA, USA). The mobile acid phase was performed using 0.5% triflouroacetic, aqueous solution (solvent A), and acetonitrile (solvent B) gradient: 0–1 min, 0% of B; 2–3 min, 5% of B; 4–20 min, 30% of B; 21–23 min, 50% of B; 24–25 min, 80% of B; 26–27 min, 100% of B; 28–30 min, 0% of B. The flow rate was 0.9 mL/min with a volume of 10 μL sample. Absorbance was measured at 270 nm [46]. A preliminary identification of the peaks resolved was performed by comparison with *t*_R_ and UV-Vis characteristic bands of known standards and literature data.

### 3.3. GC-MS Analysis of Hexane Extracts

The chemical composition of **Ij-BH** and **Ij -LH** was analyzed on Gas Chromatography-Mass Spectrometry (GC-MS) equipment, consisting of an Agilent 6890 plus gas chromatograph coupled to a simple quadrupole mass spectrometry detector, model 5972N (Agilent Technology, Santa Clara, CA, USA).

Volatile compounds were separated on an HP 5MS capillary column (25 m long, 0.2 mm i.d., with 0.3-μm film thickness). Oven temperature was set at 40 °C for 2 min, then programmed at 40–260 °C for 10 °C/min, and maintained for 20 min at 260 °C. Mass detector conditions were as follows: interphase temperature, 200 °C, and mass acquisition range, 20–550. Injector and detector temperatures were set at 250 and 280 °C, respectively. Splitless injection mode was carried out with 1 μL of each fraction (3 mg/mL solution). The carrier gas was helium at a flow rate of 1 mL/min. The identification of volatiles was performed, comparing their mass spectra with those of the National Institute of Standards and Technology (NIST) 1.7 Library and comparing these with data from the literature [47].

### 3.4. Pharmacological Activity

#### 3.4.1. Anti-Inflammatory Activity

Male ICR mice with a weight range of 25–30 g, from Envigo RMS, S.A. de C.V., were used throughout the experiments. These animals were maintained in the Bioterium of Centro de Investigación Biomédica Del Sur (CIBIS-IMSS) under a 12 h light-dark cycle and constant temperature (23–25 °C) with free access to food and water. The animals were treated under the Mexican federal regulations for care and use of laboratory animals, NOM-062-ZOO-1999 Guidelines [48], and international ethical guidelines for the care and use of experimental animals [49]; the number of animals (*n* = 5) and the intensity of the noxious stimuli utilized were the minimum necessary to demonstrate the consistent effects of the pharmacological treatments. The animal studies were approved by the Ethics Committee of the Mexican Social Security Institute (R-2020-1702-008).

Auricular inflammation was induced following the method previously described [50]. The dose evaluated for the extracts was 1.0 mg/ear. A control group received acetone as vehicle, and Indomethacin (Indo, Sigma-Aldrich, Toluca, Mexico) 1.0 mg/ear was utilized as an anti-inflammatory positive control. All treatments were dissolved in acetone and applied topically on both ears immediately after the solution of 12-*O*-tetradecanoylphorbol-13-acetate (TPA, Sigma-Aldrich, Toluca, México) as an inflammatory agent. Six hours after the administration of TPA, the animals were euthanized by cervical dislocation.

Circular sections 6 mm in diameter were taken from both the treated (t) and non-treated (nt) ears, which were weighed to determine the inflammation. The percentage of inhibition was obtained employing the expression below:

% Inhibition = [Dw control − Dw treated/Dw control] × [100]

where Dw = wt − wnt; wt is the weight of the section of the treated ear; and wnt the weight of untreated ear section.

#### 3.4.2. Antibacterial Activity

The extracts were evaluated against bacterial strains ATCC: methicillin-resistant *Staphylococcus aureus* (**Sa1**; ATCC 43330, *Staphylococcus aureus* (**Sa2**; ATCC 29213), *Staphylococcus epidermis* (**Se1**; ATCC 12228)*, Staphylococcus epidermis* (**Se2**; ATCC 35984), *Enterococcus fecalis* (**Ef**; ATCC 29212), *Escherichia coli* (**Ec1**; ATCC 25922), *Enterobacter cloacae* (**Ec2**; ATCC 700323), *Klebsiella pneumoniae* (**Kp**; ATCC 700603), *Pseudomona aeruginosa* (**Pa**; ATCC 27853), and the clinically isolated *Staphylococcus haemolyticus* (**Sh**; 1038). The strains were reseeded in antibiotic agar No. 1 (Bioxon, Mexico) for 24 h at 37 °C. The strain of clinical isolate was provided from the General Hospital of Acapulco, State of Guerrero, Mexico, to the Bacteria Bank of the Autonomous University of Guerrero (UAGro).

For the trials, cultures with 24 h of incubation (37 °C) were used and about 3–4 colonies were taken of each strain and diluted in Müeller–Hinton broth (MHb; Bioxon, Toluca, Mexico). The inoculums were adjusted using the 0.5 MacFarland scale (1.5 × 10^8^ UFC/mL). Subsequently, dilution with distilled water was performed to obtain 1 × 10^4^ UFC/mL.

The MIC of extracts was determined by the microtiter broth dilution method [51]. Briefly, the samples (50 mg/mL) were dissolved in a DMSO–water mixture (20:80), and the tested concentrations were 3.37, 6.75, 12.5, 25, 50, 100, and 200 μg/mL. The samples were added to sterile microplates of 96 wells, along with 200 μL of MHb and 2 μL of inoculum (1 × 10^4^ UFC/mL). The viability controls used were: MHb + DMSO + inoculum and MHb+ inoculum; Gentamicin (**C^+^**, 100 μg/mL; Sigma Aldrich, Mexico) was employed as the reference antibiotic. The plates were incubated at 37 °C for 24 h, and after incubation the MIC was determined by adding 30 μL of a solution (0.05%) of 3-(4,5-dimethylthiazol-2-yl)-2,5, diphenyl tetrazolium bromide (MTT, Sigma-Aldrich, Hong Kong, China) in every well, of which purple development was observed if there was viability of bacteria and colorless if there was no feasibility. All assays were performed in triplicate.

### 3.5. Statistical Analysis

For the analysis of the anti-inflammatory activity, the data were expressed as the mean ± standard error of the mean (SEM), and statistical significance was determined using an analysis of variance (ANOVA) with a confidence level of 95% (* *p* ≤ 0.05), followed by the one-tailed Dunnet test compared to Indo and the Tukey test. All analyses was performed using IBM SPSS statistics ver. 23.0 statistical program (GraphPad Software, IBM, San Diego, CA, USA).

## 4. Conclusions

This report presents the biological activity of organic extracts obtained from the bark and leaves of *I. jinicuil*. The anti-inflammatory activity tests showed moderate to good effects, with the dichloromethane extract from bark showing the highest activity, followed by the hexanic extract from leaves. Based on the findings of anti-inflammatory activity, it is possible to propose the exploration of the potential antinociceptive effect of the tested extracts, using an appropriate pharmacological model. Likewise, it was found that the three extracts from the bark of this plant have excellent antibacterial activity (primarily against methicillin-resistant *Staphylococcus aureus* and *Pseudomonas aeruginosa* strains) and this leads us to consider the possibility of extending antibacterial activity tests to a greater number of microbiological strains of clinical interest. On the other hand, it should be mentioned that, to our knowledge, this is the first approach to the phytochemical profiling of bark and leaves of *I. jinicuil*, which is consistent with the chemotaxonomic profiles reported for other species of *Inga* and suggest the presence of polyphenolic compounds, flavonoids, triterpenes, and lipid prenols, as well as aliphatic and esterified aliphatic lipids; these natural products may be responsible for both bioactivities assessed in this work. These results allow predicting a wide potential for future studies aimed at the isolation and structural characterization of compounds that might serve as molecular templates with specific biological activities. Finally, it is important to highlight that these results systematically contribute to the use in traditional Mexican medicine of a highly important sociocultural and nutritional species such as *Inga jinicuil* Schltdl & Cham. ex G. Don.

## Figures and Tables

**Table 1:** Percentages obtained from *Inga jinicuil* extracts.

Extract	*n*-Hexane (% Yield)	Dichloromethane (% Yield)	Hydroalcoholic (% Yield)
Bark extract	0.095	0.82	0.25
Leaf extract	0.95	1.02	4.65

**Table 2:** Preliminary phytochemical profile by HPLC-UV-Vis analysis of polar extracts from bark and leaves of *I. jinicuil*.

Peak	Retention Time (min)	Absorption Bands (nm)	Extract(s) *	Compound Affinity **	Ref.
1	8.46	220.4, 261.6, 294.7	■	Protocatechuic acid	Standard [15,16]
2	8.58	249.8, 273.6	●	Protocatechuic acid derivative	Standard [15,16]
3	8.58	218.1, 276.9	■	Gallic acid derivative	Standard [17,18]
4	8.66	212.2, 251.5, 352.9	□	Glycosylated Flavone. Apigenin derivative	Standard [19,20]
5	8.75	219.2, 249.8, 273.4	●	Lignane	Standard [21,22]
6	8.81	215.7, 269.8, 337.4	○□	Glycosylated Flavone. Apigenin derivative	Standard [19,20]
7	8.85	219.2, 279.3	■	Gallic acid derivative	Standard [17,18]
8	8.86	215.7, 308.9	●	Coumaric acid derivative	Standard [23]
9	8.91	207.5, 269.8, 335.1	□	Glycosylated Flavone. Apigenin derivative	[19,20]
10	9.18	249.8	○□●■	Terpene	[24]
11	9.58	245.1	○□●■	Terpene	[24]
12	9.96	209.9, 294.7, 338.6	○□	Coumarin derivative	[25,26,27]
13	10.03	276.9	○	Epigallocatechin Gallate derivative	Standard [28]
14	12.30	235.7, 266.3	○□●	Terpene	[24]
15	26.91	219.2, 273.4, 293.5	□	Vanillic acid derivative	[29,30]
16	28.01	204, 248.6	○□●■	Terpene	[24]
17	28.11	278.1	●	Epigallocatechin Gallate derivative	[28]
18	28.21	245.1, 278.1, 327.9	●	Coumarin derivative	[25,26,27]
19	28.43	201.7, 261.6	○□●■	Salicylate derivative	Standard [31]
20	28.65	200.5, 263.9	○□●■	Salicylate derivative	Standard [31]
21	28.81	263.9	○□●■	Salicylate derivative	Standard [31]

* Extracts: Bark extracts, ● (**Ij-BD**), ■ (**Ij-BHac**); Leaf extracts, ○ (**Ij-LD**), □ (**Ij-LHac**). ** Compounds were suggested by a preliminary comparison of retention time (*t*_R_) and UV-Vis bands with standards and literature data.

**Table 3:** Phytochemicals identified in hexanic extracts from the bark (**Ij-BH**) and leaves (**Ij-LH**) of *Inga jinicuil* by GC-MS.

Peak	Retention Time (min)	Molecular Weight (amu)	Extract(s) (% in the Sample) *	Compound **
22	17.80, 17.75	268.5	▲ (1.07), △ (1.18)	2-pentadecanone,6,10,14-trimethyl
23	17.80	296.5	▲ (1.07)	3,7,11,15-Tetramethyl-2-hexadecen-1-ol
24	18.55	270.5	△ (1.04)	Hexadecanoic acid, methyl ester
25	18.61	276.3	△ (0.88)	7,9-Di-tert-butyl-1-oxaspiro(4,5)deca-6,9-diene-2,8-dione
26	18.68, 18.61	270.5	▲ (3.83), △ (0.88)	Hexadecanoic acid, ethyl ester
27	20.51	296.5	▲ (11.74)	Phytol
28	20.60, 20.47	298.5	▲ (3.14), △ (0.44)	Octadecanoic acid, methyl ester
29	22.53	324.5	△ (1.35)	4,8,12,16-tetramethylheptadecan-4-olide
30	24.63	390.6	△ (0.80)	1,2-benzenedicarboxylic acid diisooctyl ester
31	27.76	380.6	△ (1.62)	15-Tetracosenoic acid, methyl ester
32	29.19	518.7	△ (1.02)	Tetradecanoic acid, 3,3a,4,6a,7,8,9,10,10a, 10b-decahydro-3a, 10a, dihydroxy-5-(hydroxymethyl)-2, 10-dimethyl-3-oxobenz [e] azulen-8-yl ester
33	29.21	410.7	▲ (5.98)	Squalene
34	30.05	408.8	▲ (12.55)	Nonacosane
35	31.95	416.7	▲ (3.74)	*β*-Tocopherol
36	32.44	436.8	▲ (16.66)	Hentriacontane
37	33.07	430.7	▲ (40.49)	α-Tocopherol
38	36.91	424.7	△ (26.74)	Lup-20 (29)-en-3-one
39	37.39	426.7	△ (16.43)	Lupeol
40	38.35	438.7	△ (38.61)	24-Methylenecycloartan-3-one
41	38.64	412.7	△ (2.27)	Stigmast-4-en-3-one
42	41.85	440.7	△ (4.99)	9,19-Cyclolanostan-3-ol,24-methylene-, (3β)-

* Extracts: Bark extract (▲ (**Ij-BH**) and leaf extract (△ (**Ij-LH**). ** Compared with the National Institute of Standards and Technology (NIST) 1.7 Library.

**Table 4:** Antibacterial activity (MIC µg/mL) of extracts from *Inga jinicuil*.

				Bacterial	Strains					
		Gram-	Positive				Gram-	Negative		
Extract	Sa1	Sa2	Se1	Se2	Sh	Ec1	Ec2	Ef	Kp	Pa
Ij-LH	>200	>200	>200	>200	>200	>200	>200	>200	>200	>200
Ij-LD	**50**	>200	**200**	>200	>200	>200	>200	>200	>200	**<3.12**
IjLHac	**50**	>200	>200	>200	>200	>200	>200	>200	>200	**<3.12**
Ij-BH	**50**	>200	**50**	>200	>200	>200	>200	>200	>200	**<3.12**
Ij-BD	**50**	>200	>200	>200	>200	>200	>200	>200	>200	**<3.12**
IjBHac	**50**	>200	>200	>200	>200	>200	>200	>200	>200	**<3.12**
C1	*	*	*	*	*	*	*	*	*	*
C2	*	*	*	*	*	*	*	*	*	*
C+	--	--	--	--	--	--	--	--	--	--

**Sa1**: methicillin-resistant *S. aureus*; **Sa2**: *S. aureus*; **Se1**: *S. epidermis*; **Se2**: *S. epidermis*; **Sh**: clinically isolated *S. haemolyticus*; **Ec1**: *E. coli***Ec2**: *E. cloacae*; **Ef**: *E. fecalis*; **Kp**: *K. pneumoniae*; **Pa**: *P. aeruginosa*; **C1** and **C2**: controls of viability (*: bacterial growth); **C+**: positive control (Gentamicine 100 μg/mL; --: not bacterial growth).

## Data Availability

Data is contained within the article and Supplementary Material.

## Supplementary Materials

The following are available online at https://www.mdpi.com/article/10.3390/plants11060794/s1, Figure S1: UV-spectra of the main compounds of (Ij-BD) *I. jinicuil*; Figure S2: UV-spectra of the main compounds of (Ij-BHac) *I. jinicuil*; Figure S3: UV-spectra of the main compounds of (Ij-LD) *I. jinicuil*; Figure S4: UV-spectra of the main compounds of (Ij-LHac) *I. jinicuil*.
