# Supplementary material for: Preliminary Phytochemical Profile and Bioactivity of Inga jinicuil Schltdl & Cham. ex G. Don
Source: Plants (Basel). 2022 Mar 17;11(6):794. doi: 10.3390/plants11060794 (PMC8953309; doi:10.3390/plants11060794)
Supplement: Supplementary file 1 [file plants-11-00794-s001.zip › plants-1623027-supplementary.pdf]

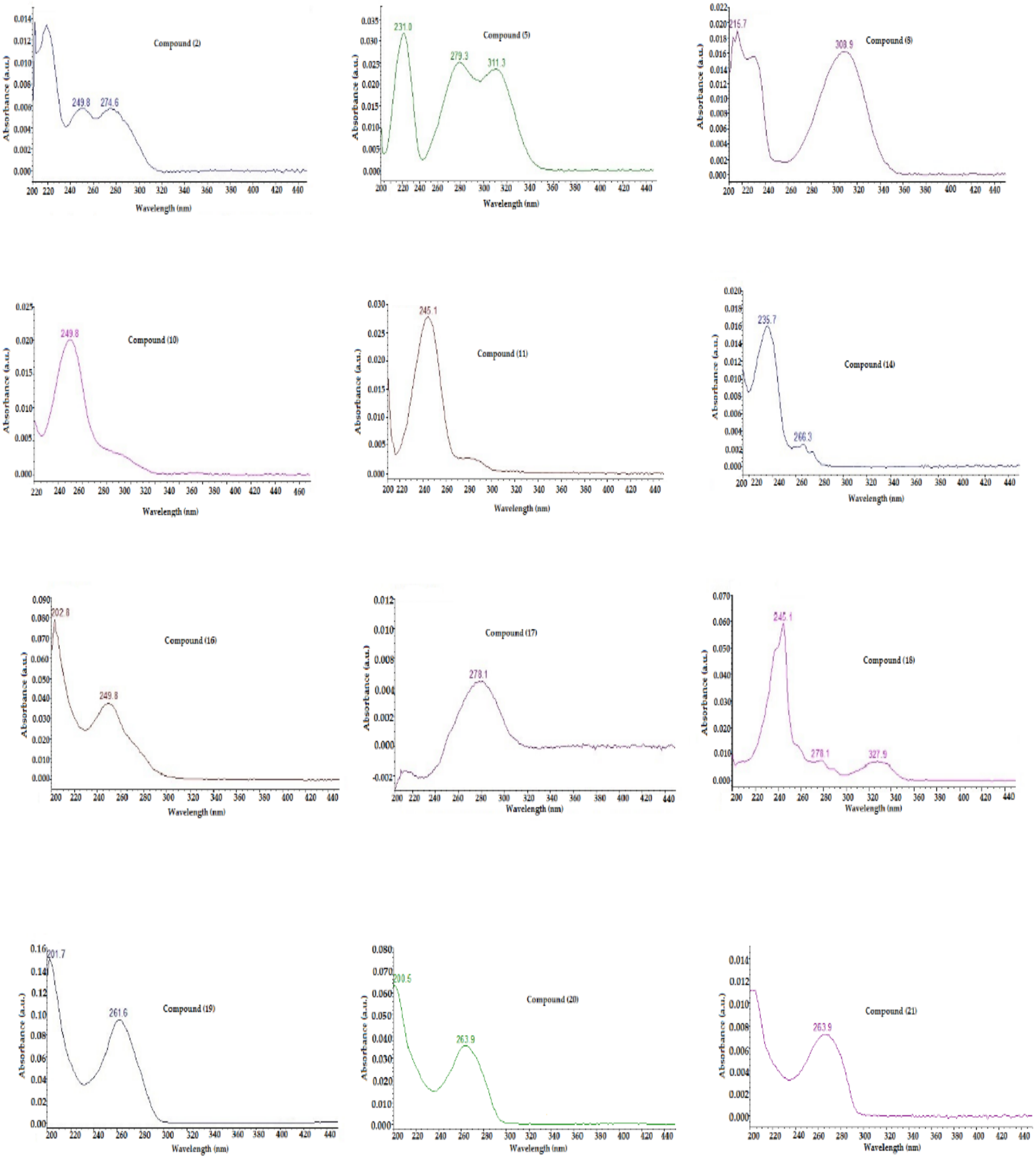

Figure S1: UV-spectra of the main compounds of (Ij-BD) *I. jinicuil*

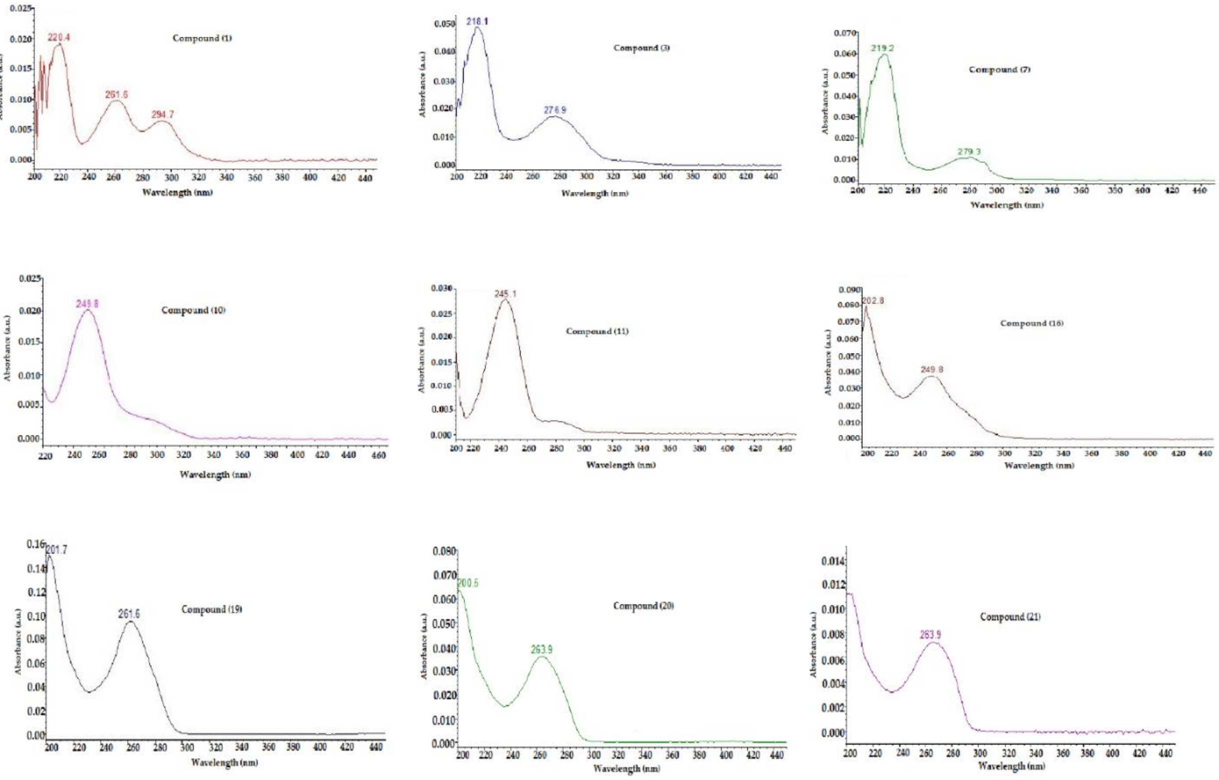

Figure S2: UV-spectra of the main compounds of (Ij-BHac) *I. jinicuil*

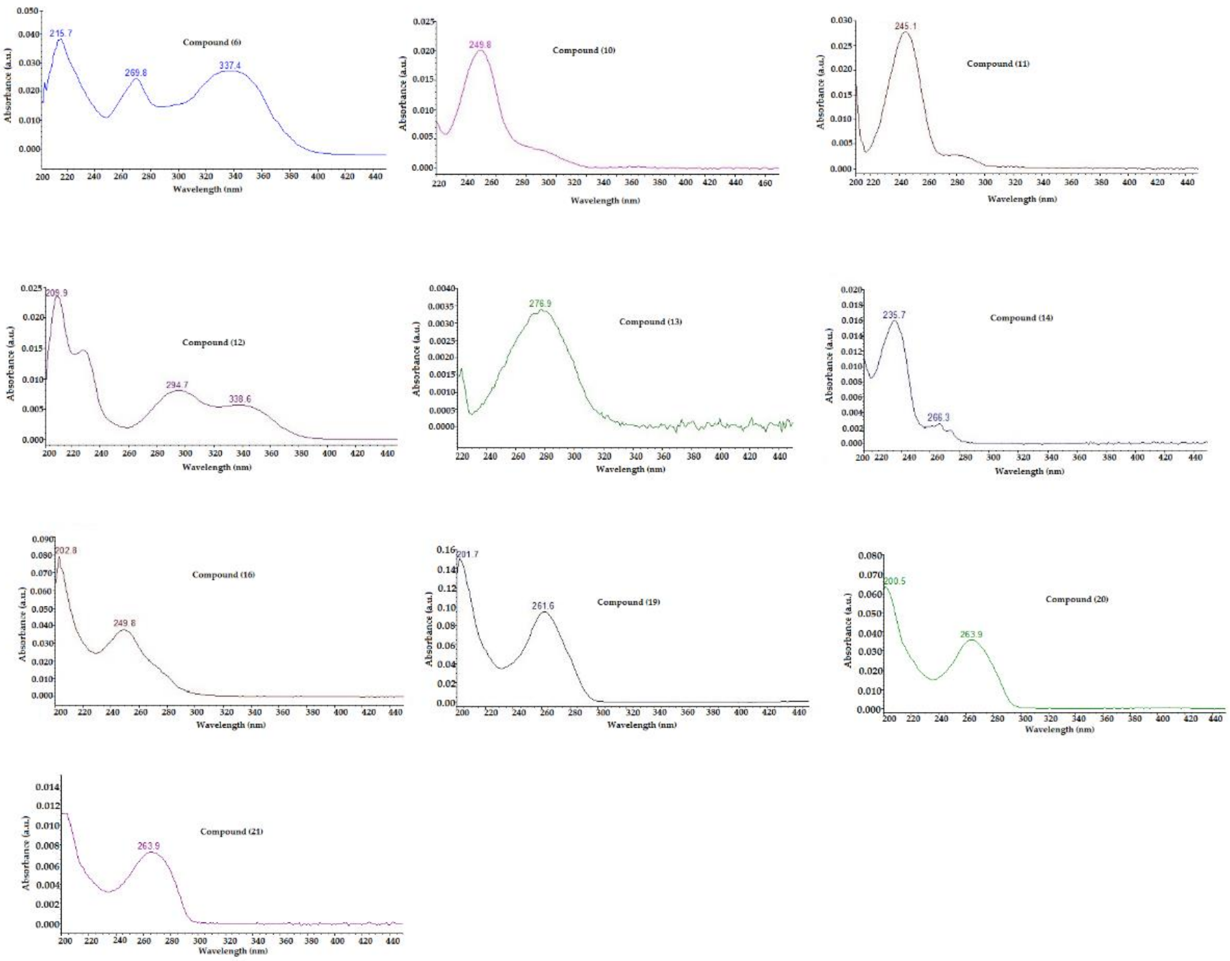

Figure S3: UV-spectra of the main compounds of (Ij-LD) *I. jinicuil*

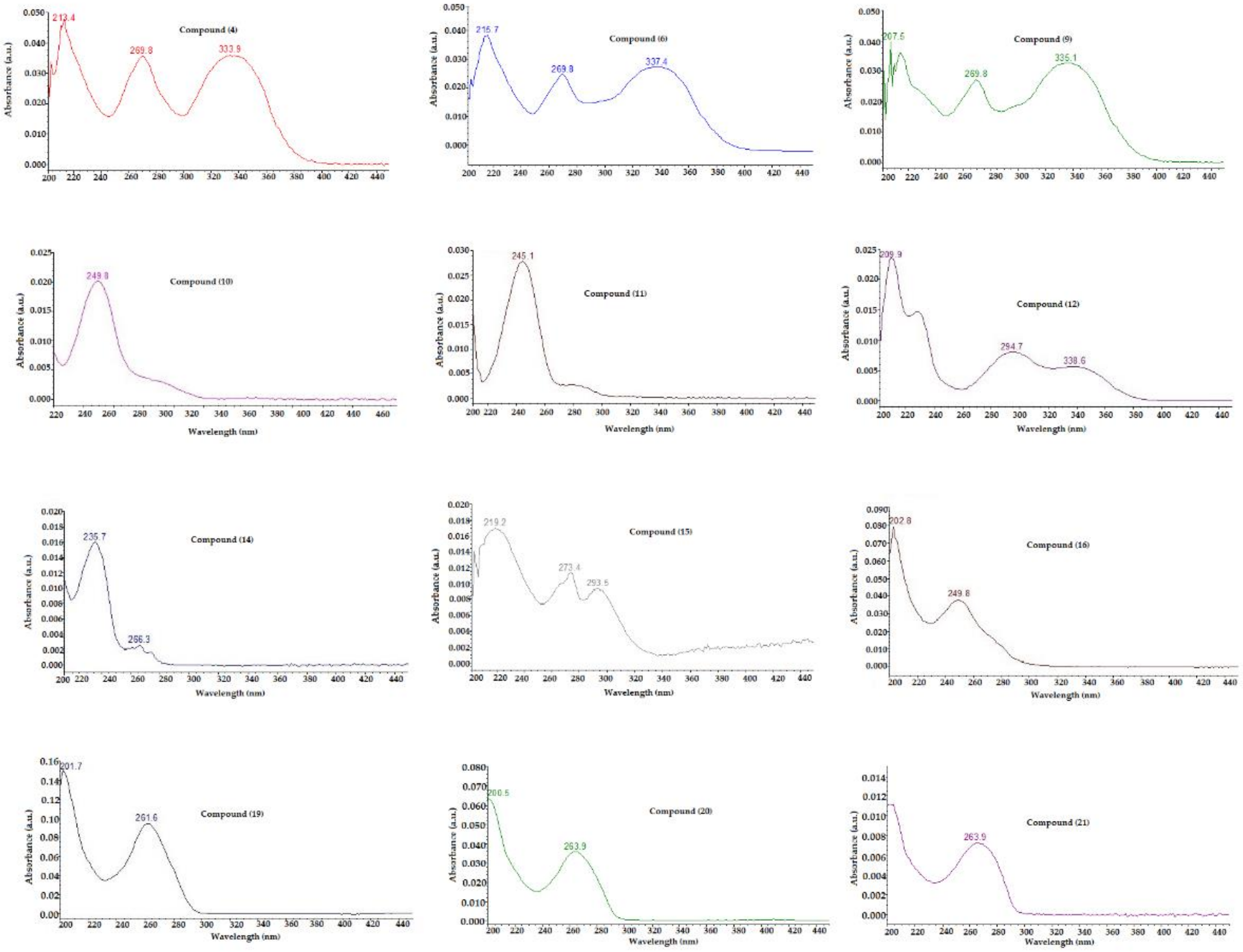

Figure S4: UV-spectra of the main compounds of (Ij-LHac) *I. jinicuil*
